# Comparison of Antimicrobial Activity against *Porphyromonas gingivalis* between Advanced Platelet-Rich Fibrin and Injectable Platelet-Rich Fibrin

**DOI:** 10.1155/2023/9194868

**Published:** 2023-03-27

**Authors:** Thuy Anh Vu Pham

**Affiliations:** ^1^Division of Odonto-Stomatology, School of Medicine, Ho Chi Minh City, Vietnam; ^2^Vietnam National University, Ho Chi Minh City, Vietnam

## Abstract

Platelet-rich fibrin (PRF) obtained via low-speed centrifugation has antimicrobial properties. This study was conducted to evaluate the effectiveness of advanced platelet-rich fibrin plus (A-PRF+) and injectable platelet-rich fibrin (I-PRF), obtained from patients with different periodontal states, against *Porphyromonas gingivalis*. A-PRF+ and I-PRF samples were obtained from venous blood of 60 subjects divided equally into three groups: periodontitis, gingivitis, and healthy gingiva groups. The antibacterial experiments evaluated biofilm inhibition, mature biofilm impact, and time-kill kinetics. The percent reduction in biofilm-growing and mature biofilm bacteria ranged from 39% to 49% and 3% to 7%, respectively. In the time-kill kinetics assay, PRF from the periodontitis group was more effective as an antimicrobial than that from the gingivitis and healthy gingiva group (*p* < 0.001); I-PRF was more effective than A-PRF+ (*p* < 0.05) and both of them showed peak antibacterial activity after 12 h of exposure. Both A-PRF+ and I-PRF exhibited antibacterial properties against *P. gingivalis*, but I-PRF appeared to be more effective. The PRF obtained from the different groups appeared to have different degrees of antimicrobial efficacy.

## 1. Introduction

Periodontal disease is an inflammatory disease of the tooth-supporting tissues, e.g., gingiva, periodontal membrane, and alveolar bone, induced by oral microbes, dental plaque, or dental biofilm [1]. The presence of certain bacterial species, isolated as five microbial complexes found together in the subgingival biofilm, is implicated in the occurrence of periodontal disease. Among these bacteria, *Porphyromonas gingivalis* (*P. gingivalis*), which is able to combine with other strains of bacteria, has previously been shown to play a crucial role in periodontal pathology [2]. *P. gingivalis* is one of the most prevalent bacteria in periodontitis and is also recognized as a keystone pathogen in peri-implantitis, a periodontal disease, which is why in the treatment of periodontal disease more and more antimicrobial strategies focus on restricting the microbial growth and activity of this bacterium [3]. *P. gingivalis* is a Gram-negative oral anaerobe and is considered a main etiological factor in periodontal diseases, producing several virulence factors and extracellular proteases such as lipopolysaccharide, fimbria, and gingipain that result in destruction of the periodontal tissue [4].

The main therapeutic strategies of periodontal treatment are mechanical debridement by scaling and root planing, and periodontal surgery to remove dental plaque, and hopefully all the associated bacteria at the sites of infection. However, one of the major problems associated with surgical procedures and wound-healing processes is the potential risk of bacterial contamination and infection [5]. Thus, control of infections plays a key role in successful wound healing and avoiding chronic conditions. Because of the adverse effects stemming from the development of resistant bacteria, the use of postoperative systemic antibiotics following dental surgery has become controversial [6]. Recently, platelet concentrates have been used for the successful treatment of periodontal disease based on their regenerative potential and anti-inflammatory action and reported activity against various bacteria strains [2], which could make them an alternative to systemic antibiotics. PRF is a second-generation platelet concentrate comprising a complex network of microfibrins with entrapped platelets and leukocytes [7]. Notably, the leukocytes in PRF are known to exhibit antimicrobial activity [2].

The use of low-speed centrifugation to obtain new types of PRF has previously been explored, having the advantage of producing a high number of cells such as leukocytes [8]. I-PRF (injectable PRF) is available in injectable form and coagulates a few minutes after injection [2] while A-PRF+ (advanced PRF) has shown superior properties when compared with standard PRF [8]. These two types of PRF have great potential for further clinical application because they are simple to prepare and use and do not require the addition of anticoagulants [6] and also have an enriched antimicrobial content [7]. A-PRF has outstanding advantages, including the increased release of proteins and growth factors, proliferation of fibroblasts, its ability to migrate, and expression of growth factors [8]. I-PRF contains various components such as antimicrobial proteins, complement binding proteins, and antimicrobial peptides [7].

It has been demonstrated that the quality of PRF depends on several patient-related factors, especially their periodontal condition [9]. However, this issue has not been given proper attention in previous studies, in which only subjects without periodontal problems were selected to provide samples [5, 7]. An *in vitro* study reported that periodontal diseases including periodontitis with evidence of inflammatory responses could alter the amount of growth factors released by PRF [10]. To clarify this issue, the subjects recruited for our current study were expanded to include patients with gingivitis and periodontitis, in addition to subjects with healthy gingiva. This wider sampling was expected to provide novel data.

Thus, the aim of the present study was to provide *in vitro* evidence of the antimicrobial properties of new generations of PRF, including I-PRF and A-PRF+ that were obtained from patients with different periodontal conditions, against *P. gingivalis*.

## 2. Subjects and Methods

### 2.1. Participant Selection

Sixty patients aged 20–65 years who attended the Department of Periodontology, Odonto-Maxillo-Facial Hospital in Ho Chi Minh City and volunteered to participate in the study were included in our study sample. All were nonsmokers with no history of infection or any antibiotic or anti-inflammatory use for at least 3 months before the study. None of them gave a history of any anticoagulant or immunosuppressive therapy that might interfere with the natural coagulation process nor did they report any bleeding or clotting disorders. All of them were examined and diagnosed by a periodontist prior to being divided into three groups of participants (20 patients for each): Group 1: patients without gingival diseases such as gingival bleeding and swelling, whose periodontal pocket depth was under 3 mm. Group 2: patients who had severe gingivitis with gingival bleeding around over a half of their dentition. Group 3: patients with moderate or severe chronic periodontitis according to the classification of the American Association of Periodontology, 2017 [11]. All patients gave written informed consent before beginning the study. The study protocol and consent procedure were approved by the Ethics Committee of Ho Chi Minh Odonto-Stomatology Hospital, Vietnam (no. 536/BVRHM), and were conducted in full accordance with the World Medical Association Declaration of Helsinki.

### 2.2. Sample Preparation

Twenty milliliters of blood were collected from each patient and placed immediately in the Dou Quattro Choukroun PRF system (designed by PROCESS for PRF ®, Nice, France). For the A-PRF+, 10 ml of blood was placed in a sterile plain glass-based vacuum tube and centrifuged according to the A-PRF+ mode with a preset rotation speed of 1,300 rpm for 8 minutes [12]. To obtain I-PRF, the blood tube was centrifuged at 700 rpm for 3 min (60 × g) [13]. The A-PRF+ clot was then washed and sectioned, and its volume was measured by placing it in an Eppendorf containing 0.5 ml of sterile 1X PBS and marking the rise in the liquid level. The obtained A-PRF and I-PRF were immediately used in antimicrobial tests.

### 2.3. Tests Performed

A sterile inoculating loop was used to transfer an inoculum of *P. gingivalis*, which was cultured from subgingival plaque of patients with periodontitis in our previous study [14], onto Petri dishes that contained Wilkins–Chalgren Anaerobe Agar (Oxoid LP0011B) supplemented with 5% laked sheep blood (culture medium). Petri dishes were incubated under anaerobic conditions with Gaspak kits (Merk, USA) at 37°C for 7 days. Sufficient inoculum was added to Petri dishes until the turbidity equaled 0.5 McFarland. This prepared a bacterial suspension and was used in the subsequent experimental assays of antimicrobial activity.

#### 2.3.1. Biofilm Inhibitory Assay

For the A-PRF+ experiment, overnight bacterial cultures were prepared in TSB medium at 37°C. Aliquots of 200 *μ*l A-PRF+ 40% and bacterial inoculum were placed in sterile polystyrene test tubes. The control tubes contained only culture medium. All of the tubes were incubated anaerobically for 24 hours at 37°C. Then, the nonadherent cells were washed twice with PBS while the adherent cells were fixed by adding methanol for 15 minutes before staining with crystal violet 0.1% for 5 minutes. Distilled water was used to wash away the excess stain, then, the test tubes were air-dried before adding 200 *μ*l ethanol 95%. The quantity of biofilm was determined by measuring absorbance in a microplate reader at the wavelength of 610 nm.

The procedure for the I-PRF experiment was similar to that for A-PRF+, with aliquots of 150 *μ*l I-PRF and inoculum placed in sterile 96-well polystyrene plates that had been pretreated by the manufacturer. The control subgroup was incubated in culture medium. The plates were incubated anaerobically for 24 hours at 37°C prior to being washed twice with PBS and then fixed with methanol for 15 minutes before staining with crystal violet 0.1% for 5 minutes. Distilled water and an air-dryer were used, and 200 *μ*l ethanol 95% was added. A microplate reader was used to measure the absorbance in each well at the wavelength of 610 nm.

#### 2.3.2. Mature Biofilm Impact Assay

To visualize the impact of A-PRF+ and I-PRF on the mature biofilm, the test tubes or plates containing only bacterial inoculum were incubated anaerobically for 48 hours to allow a mature bacterial biofilm to form. Then, the culture solution was removed and 200 *μ*l A-PRF+ 40% was added to the test tubes and 150 *μ*l I-PRF was added to the test plates. All of the tubes and plates were incubated anaerobically for 4 hours at 37°C before they were washed twice with PBS and then fixed with methanol for 15 minutes before staining with crystal violet 0.1% for 5 minutes. Distilled water was used to wash away the excess stain, then, the test tubes were air-dried before adding 200 *μ*l ethanol 95%. The quantity of biofilm was determined by measuring absorbance in a microplate reader at the wavelength of 610 nm.

#### 2.3.3. Time-Kill Kinetics Assay

The time-kill kinetics assay was conducted using 800 *μ*l A-PRF+ or I-PRF 40% in culture medium placed in sterile polystyrene test tubes. Culture medium without PRF was placed in the control tubes. An inoculum of 5.0 × 10^5^ CFU/mL was also added and anaerobically incubated at 37°C. At 0, 3, 6, 12, 24, 36, and 48 hours of incubation, serial dilutions were performed (down to 10^−4^) and 50 *μ*l was plated onto Wilkins–Chalgren agar plates. These were incubated in an anaerobic chamber for a further 24 hours and colony counting was performed.

### 2.4. Statistical Analysis

All experiments were performed in triplicate and repeated at least three times. Results are reported as the mean ± standard deviation. Analysis was performed using either one-way analysis of variance or repeated measures analysis. *P* values <0.05 were considered to be significant. For statistical analysis, SPSS v.23 software (IBM, New York City, NY, USA) was used.

## 3. Results

### 3.1. Demographic Data

The demographic characteristics of the participants are presented in Table 1. A total of 60 people compliant with our research standards were divided equally into three groups depending on their periodontal condition, which included healthy gingiva, gingivitis and periodontitis as mentioned in the Methods section. The mean age of the healthy gingiva, gingivitis, and periodontitis groups was 23.7 ± 5.2, 33.0 ± 7.4, and 50.9 ± 13.3, respectively, with significant differences between groups (*p* < 0.001). The gender ratio in the three groups did not differ significantly (*p* > 0.05).

### 3.2. Biofilm Inhibitory Assay

Antimicrobial efficacy was demonstrated by the percentage reduction in the number of bacteria in the test groups compared to that in the control group, as shown in Table 2.

No differences in antibacterial effect on biofilm formation of *P. gingivalis* were found between the various A-PRF+ samples derived from the blood of different patient groups. The percent reduction in bacterial number in the periodontitis, gingivitis, and healthy gingiva groups was 42 ± 9%, 41 ± 9%, and 39 ± 9%, respectively. The difference between groups was not significant (*p* > 0.05). Similarly, the percent reduction following exposure to the I-PRF samples was 49 ± 8%, 47 ± 10%, and 45 ± 10% in the periodontitis, gingivitis, and healthy gingiva groups, respectively. Likewise, there was no significant difference between the groups (*p* > 0.05).

In the periodontitis group, the percentage reduction in bacterial number following exposure to A-PRF+ was significantly lower than following exposure to I-PRF (*p* < 0.05). Overall, there was a trend for a lower percent reduction following exposure to A-PRF+ in the gingivitis and healthy gingiva groups relative to I-PRF, but the differences were not significant (*p* > 0.05).

### 3.3. Mature Biofilm Impact Assay

The antimicrobial impact of PRF on mature biofilm of *P. gingivalis* was expressed as the percent reduction in bacterial number in the test groups compared to that in the control group, as shown in Table 3.

No differences in antibacterial effect on mature biofilm of *P. gingivalis* were found between the various A-PRF+ samples that were derived from the blood of different patient groups (3 ± 5%, 3 ± 6%, and 5 ± 9%, in the periodontitis, gingivitis, and healthy gingiva groups, respectively; *p* > 0.05). Similarly, the percent reduction following exposure to I-PRF was 5 ± 5%, 5 ± 5%, and 7 ± 8% in the periodontitis, gingivitis, and healthy gingiva groups, respectively (*p* > 0.05).

There was a trend for a lower percentage reduction following exposure to A-PRF+ from all groups compared with exposure to I-PRF; however, the differences were not significant (*p* > 0.05).

### 3.4. Time-Kill Kinetics Assay

The percent reduction in bacterial colony number in the test groups compared to that in the control group at the corresponding time-points (3, 6, 12, 24, 36, and 48 hours) under the influence of A-PRF+ and I-PRF is presented in Table 4.

**Figure 1:**
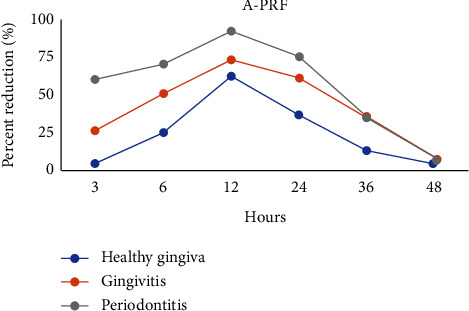
The percent reduction in the number of bacterial colonies compared to the control under the influence of A-PRF+ from the three groups at 3, 6, 12, 24, 36, and 48 h.

**Figure 2:**
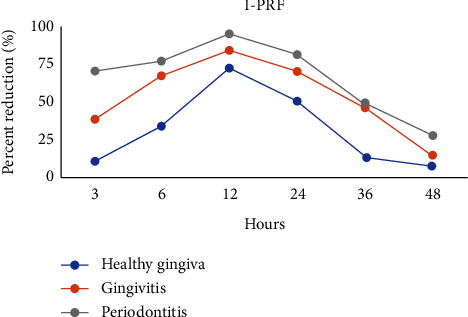
The percent reduction in the number of bacterial colonies compared to the control under the influence of I-PRF from the three groups at 3, 6, 12, 24, 36, and 48 h.

The percent decrease in bacterial colony number varied significantly among the healthy gingiva, gingivitis, and periodontitis groups (*p* < 0.001). In particular, at 3 h, 6 h, 12 h, 24 h, and 36 h, the percent reduction was highest in the periodontitis group and lowest in the healthy gingiva group. However, there was no significant difference at 48 h (*p* > 0.05). These data are depicted in Figures 1 and 2.

Regarding the antimicrobial efficacy of A-PRF+ and I-PRF, there was a higher percent reduction following exposure to I-PRF than A-PRF+. In the group of subjects with healthy gingiva, the difference was not significant. In the gingiva group, significant differences were found at 6 h, 12 h, 24 h, and 36 h while in the periodontitis group the difference was also significant at 3 h (*p* < 0.05).

As shown in Figures 1 and 2, the percent reduction of microbial colonies changed over time, increasing from 3 h to 6 h and peaking at 12 h, before gradually decreasing to the end of the observation period.

## 4. Discussion

In the current study, the mean age of patients was the highest in the periodontitis group and the lowest in the healthy gingiva group, indicating that the severity of periodontal diseases increases with aging; in other words, age is a contributing factor related to periodontal disease, as indicated in a previous study [15]. In the current study, the mean age of periodontitis patients was around 50 years, which was significantly higher than that of the remaining groups. This result was relatively consistent with the previous study, which showed that patients in their fourth decades of life witnessed a remarkable increase in the prevalence of severe periodontitis [15]. Therefore, the elderly population's oral health should be given more attention.

Autologous platelet concentrates were initially used to stimulate tissue healing. PRF has recently been of particular interest to researchers and clinicians because of its high clinical applicability in the treatment of bony defects, dental implant surgery, and postextraction healing and its ability to reduce the rate of postsurgery complications [6]. Moreover, it has been suggested that PRF, which was found to have antimicrobial properties thanks to its leukocyte component [2], could be used as a supportive agent and even as an alternative to antibiotics, whose side effects could lead to the development of antibiotic-resistant bacteria [5, 6]. In the current study, two kinds of PRF (A-PRF+ and I-PRF) were obtained following the “low-speed concept” for blood centrifugation, whereby lower centrifugation speeds were shown to result in higher amounts of growth factors and higher numbers of cells including leukocytes [10], which play an important role in PRF's antibacterial mechanism [2]. A recent study found that leukocytes are influenced by the roles of platelets to modulate their behaviors, and enhance their ability to phagocytose and kill microorganisms by triggering different types of signaling pathways [16]. The advantages of utilizing PRF as an autologous source of growth factors in comparison to other allograft or xenograft sources are that concerns regarding disease transmission and adverse immunogenic reactions are eliminated [10]. The characteristics of platelet concentrate depend a lot on the generation step. By that time, the literature gave more modification to promote the regenerative potential of this biological material and focused on the analysis of each PRF protocol [17, 18]. In this current study, we generated A-PRF+ and I-PRF with the initial protocol, and primarily, to obtain the highest volume of leukocytes and platelets for I-PRF, we applied the harvesting method of Miron et al., in which collected the cell accumulating just above the red blood cell layer [19]. However, we had not tried horizontal centrifugation, which generated, according to Fujioka-Kobayashi et al., higher concentrations than those caused by routine fixed-angle centrifugation [20]. It is potential for future research if the last one can express a higher antimicrobial potential.

In another hand, in the context of the potential development of antibiotic-resistant bacteria, and the fact that *P. gingivalis* has been found to have an efficient mechanism for the transfer of resistance factors [6, 21], the antibacterial properties of PRF are particularly beneficial. After *Aggregatibacter actinomycetemcomitans*, *P. gingivalis* is regarded as the periodontal pathogen that has received the most research. Within this family of asaccharolytic, intermediate, and highly saccharolytic species, they were instantly linked to periodontal disease. Gingivalis can damage the endothelium and gingival mucosal epithelial cells. As a result of the bacterium using fimbriae, this fact can be found both above and inside the epithelial cells [22]. In this study, we used the bacteria sample directly cultured from subgingival plaque to investigate the antimicrobial of platelet concentrates instead of commercial strains. According to Yang et al., the results can be readily confirmed by and compared to those of other laboratories using these thoroughly researched bacterial strains. Yet, after several passages in the lab, these strains may lose some of their harmful characteristics. Therefore, to examine these plasma preparations' antibacterial properties, it could be necessary to use recent clinical isolates [5]. A-PRF+ and I-PRF, two of the latest kinds of PRF, have been demonstrated to have superior properties over other types of PRF [16]. However, few studies have been conducted to compare the antimicrobial features of these two kinds of PRF, which is why our study was carried out using a biofilm inhibitory assay, mature biofilm impact assay, and time-kill kinetics assay.

In the current study, both kinds of PRF were found to significantly reduce *P. gingivalis* biofilm formation in the biofilm inhibitory assay, with the percent reduction in bacterial number ranging from 39% to 49%. A study by Jasmine et al. also found a significant percent reduction in biofilm-producing bacteria but was conducted on other bacterial strains (*S. epidermis* and *S. aureus*) [7]. Meanwhile, the mature biofilm impact assay in our study found no clear impact of A-PRF+ and I-PRF on mature biofilm of *P. gingivalis*, with a percent reduction of only around 3% to 7%. A previous study showed the remarkable resistance of biofilm-growing and mature biofilm bacteria to antibiotic therapy and phagocytosis [23]. Mature biofilms were demonstrated to be more resistant to host immune defenses and the action of antibacterial agents [24]. This may be why PRF in this study had a greater impact on bacteria at the stage of forming biofilm rather than those at the stage of producing mature biofilm. Interestingly, no difference between A-PRF+ and I-PRF was found in these two assays, although I-PRF was found to contain more growth factors in a previous study [2]. Specifically, it was reported that the wide range of inhibitory and bactericidal activity of I-PRF is due to it containing platelets, fibrin, fibronectin, thrombin, HBD-3 peptide (antimicrobial peptide) myeloperoxidase, and white blood cells [7].

In the time-kill kinetics assay, I-PRF and A-PRF+ had a positive antimicrobial impact on *P. gingivalis* during the first 48 h, with a peak at 12 h, when the percent reduction was as high as 92–95% in the periodontitis group. A previous study also showed that PRF demonstrated inhibitory activities against *P. gingivalis* for up to 36 h [5]. The time-kill assay in the latter study was assessed after 50 h while our assay was assessed after 3, 6, 12, 24, 36, and 48 h. Our study results do not support previous research that found that the effects of PRF only lasted for 48 hours, during which growth factors were released at the highest concentration [2]. In our study, I-PRF had a significantly better antibacterial effect than A-PRF in the periodontal disease groups, consistent with recent studies [2, 25]. I-PRF, which was obtained using a slower and shorter centrifugation spin, contains a higher number of regenerative cells and a higher concentration of growth factors than A-PRF [2]. In the current study, A-PRF+ was obtained after centrifuging blood samples at 1,300 rpm for 8 minutes while I-PRF was obtained after a lower and shorter centrifugation spin, at 700 rpm for 3 minutes. Although I-PRF has similar properties to A-PRF, it is obtained in an injectable liquid form without forming a PRF membrane. The liquid form of platelet concentrates has better antimicrobial ability than the membrane form [8]. In a previous study, I-PRF had the maximum antibacterial activity followed by PRP, and the lowest antibacterial activity was shown by PRF when tested against *P. gingivalis* [8].

To the best of our knowledge, previous studies have rarely used PRF obtained from patients with periodontal diseases [2, 5, 7], instead collecting blood samples from healthy donors [5, 7]. In other words, patients who had periodontal problems were excluded from the study sample, which is a limitation of those studies. It is worth mentioning that patient-related factors such as chronic periodontitis may affect the quality of PRF [9], as although there were no significant differences in cell proliferation between the healthy and periodontitis groups, there was a trend for increased cell proliferation in the periodontitis group [9]. This could be because patients with chronic periodontitis have increased systemic levels of proinflammatory cytokines and growth factors, which may affect the quantity and quality of the PRF [10]. Moreover, possible connections between periodontitis and systemic diseases were demonstrated by evidence of systemic inflammatory responses that could activate platelets to release growth factors, and possibly with inflammation, where the healing response may be downregulated, with decreased amounts of growth factors from PRF [10]. To date, whether the quality of PRF is significantly dependent on periodontal disease status remains a controversial issue that needs further investigation. Although in a previous study, the impact of periodontal condition on PRF as an autologous source of growth factors was not confirmed, the authors noted their small number of subjects and large standard errors [10]. Our study is thus the first to evaluate and compare three groups of patients with healthy gingiva, gingivitis, and periodontitis.

According to the results of our time-kill kinetics assay, PRF from periodontitis patients was more effective against *P. gingivalis* than that from the gingivitis or healthy gingiva groups. On the contrary, no significant differences were found between groups in the biofilm inhibitory assay or mature biofilm impact assay. Thus, further research is needed on patient-related factors in relation to PRF to reach a clearer conclusion. Although a strength of our study was that it is the first to assess classified subjects based on their periodontal condition, the nature and features of PRF that were collected from different subjects such as their proinflammatory cytokine content and growth factor release were beyond the scope of this study. These aspects should be addressed in future studies.

## 5. Conclusions

PRF not only stimulates the healing process of periodontal tissue but also has significant antimicrobial efficacy against *P. gingivalis*. The PRF samples that were obtained from patients with different periodontal disease status appeared to have different antimicrobial efficacy. Specifically, the PRF from the periodontitis group was more effective than that from the gingivitis group, while PRF from the healthy gingiva group was the least effective. Of the two kinds of PRF used in the current study, I-PRF had better antibacterial properties than A-PRF+.

## Figures and Tables

**Table 1:** Demographic data.

Groups	Age, mean ± SD	*Gender, N (%)*		*p* value^*∗*^
		Male	Female	
Healthy gingiva	23.7 ± 5.2	11 (55)	9 (45)	0.812
Gingivitis	33.0 ± 7.4	12 (60)	8 (40)	
Periodontitis	50.9 ± 13.3	13 (65)	7 (35)	
*p* value^*∗∗*^	<0.001			

^*∗*^Chi-square test; ^*∗∗*^One-way ANOVA test. Statistical significance was set at *p* < 0.05. SD, standard deviation.

**Table 2:** Biofilm inhibitory assay: the percent reduction in the number of bacteria (%) compared to the control under the influence of different blood-derived products (A-PRF+ and I-PRF) from the three groups.

Groups	A-PRF+	I-PRF	*p* values^*∗*^
Healthy gingiva	0.39 ± 0.09	0.45 ± 0.10	0.053
Gingivitis	0.41 ± 0.09	0.47 ± 0.10	0.050
Periodontitis	0.42 ± 0.09	0.49 ± 0.08	0.021
*p* value^*∗∗*^	0.572	0.575	

Data are presented as the mean ± SD (SD, standard deviation). ^*∗*^Comparison between A-PRF+ and I-PRF, two-sample*t* test; ^*∗∗*^comparison among three patient groups, one-way ANOVA. Statistical significance was set at *p* < 0.05.

**Table 3:** Mature biofilm impact assay: the percent reduction in the number of bacteria (%) compared to the control under the influence of different blood-derived products (A-PRF+ and I-PRF) from the three groups.

Groups	A-PRF+	I-PRF	*p* values^*∗*^
Healthy gingiva	0.05 ± 0.09	0.07 ± 0.08	0.467
Gingivitis	0.03 ± 0.06	0.05 ± 0.05	0.169
Periodontitis	0.03 ± 0.05	0.05 ± 0.05	0.295
*p* value^*∗∗*^	0.395	0.337	

Data are presented as the mean ± SD (SD, standard deviation). ^*∗*^Comparison between A-PRF+ and I-PRF, two-sample*t* test; ^*∗∗*^comparison among three patient groups, one-way ANOVA. Statistical significance was set at *p* < 0.05.

**Table 4:** Time-kill kinetics assay: the percent reduction in the number of bacterial colonies compared to the control under the influence of different blood-derived products (A-PRF+ and I-PRF) from the three groups at 3, 6, 12, 24, 36, and 48 h.

	Groups	Healthy gingiva	Gingivitis	Periodontitis	*p* values^*∗∗*^
3 h	A-PRF+	0.05 ± 0.19	0.26 ± 0.19	0.60 ± 0.17	<0.001
	I-PRF	0.10 ± 0.23	0.39 ± 0.34	0.71 ± 0.12	<0.001
	*p* value^*∗*^	0.412	0.171	0.021	

6 h	A-PRF+	0.25 ± 0.18	0.51 ± 0.23	0.71 ± 0.10	<0.001
	I-PRF	0.34 ± 0.14	0.67 ± 0.13	0.77 ± 0.07	<0.001
	*p* value^*∗*^	0.071	0.008	0.039	

12 h	A-PRF+	0.62 ± 0.23	0.73 ± 0.14	0.92 ± 0.05	<0.001
	I-PRF	0.73 ± 0.25	0.84 ± 0.05	0.95 ± 0.02	<0.001
	*p* value^*∗*^	0.140	0.002	0.032	

24 h	A-PRF+	0.37 ± 0.25	0.61 ± 0.08	0.75 ± 0.08	<0.001
	I-PRF	0.51 ± 0.32	0.70 ± 0.08	0.81 ± 0.04	<0.001
	*p* value^*∗*^	0.117	0.001	0.011	

36 h	A-PRF+	0.14 ± 0.30	0.36 ± 0.10	0.36 ± 0.18	<0.001
	I-PRF	0.14 ± 0.32	0.46 ± 0.11	0.49 ± 0.14	<0.001
	*p* value^*∗*^	0.997	0.004	0.018	

48 h	A-PRF+	0.05 ± 0.20	0.08 ± 0.19	0.07 ± 0.21	0.888
	I-PRF	0.08 ± 0.18	0.15 ± 0.19	0.28 ± 0.40	0.089
	*p* value^*∗*^	0.546	0.220	0.045	

Data are presented as the mean ± SD (SD, standard deviation). ^*∗*^Comparison between A-PRF+ and I-PRF, two-sample*t* test; ^*∗∗*^comparison among three patient groups, one-way ANOVA. Statistical significance was set at *p* < 0.05.

## Data Availability

All the data used to support the findings of this study are included within the article.
